# Early Executive Function at Age Two Predicts Emergent Mathematics and Literacy at Age Five

**DOI:** 10.3389/fpsyg.2017.01706

**Published:** 2017-10-12

**Authors:** Hanna Mulder, Josje Verhagen, Sanne H. G. Van der Ven, Pauline L. Slot, Paul P. M. Leseman

**Affiliations:** Department of Special Education: Cognitive and Motor Disabilities, Utrecht University, Utrecht, Netherlands

**Keywords:** executive function, two-year-olds, mathematics, literacy, kindergartners

## Abstract

Previous work has shown that individual differences in executive function (EF) are predictive of academic skills in preschoolers, kindergartners, and older children. Across studies, EF is a stronger predictor of emergent mathematics than literacy. However, research on EF in children below age three is scarce, and it is currently unknown whether EF, as assessed in toddlerhood, predicts emergent academic skills a few years later. This longitudinal study investigates whether early EF, assessed at two years, predicts (emergent) academic skills, at five years. It examines, furthermore, whether early EF is a significantly stronger predictor of emergent mathematics than of emergent literacy, as has been found in previous work on older children. A sample of 552 children was assessed on various EF and EF-precursor tasks at two years. At age five, these children performed several emergent mathematics and literacy tasks. Structural Equation Modeling was used to investigate the relationships between early EF and academic skills, modeled as latent factors. Results showed that early EF at age two was a significant and relatively strong predictor of both emergent mathematics and literacy at age five, after controlling for receptive vocabulary, parental education, and home language. Predictive relations were significantly stronger for mathematics than literacy, but only when a verbal short-term memory measure was left out as an indicator to the latent early EF construct. These findings show that individual differences in emergent academic skills just prior to entry into the formal education system can be traced back to individual differences in early EF in toddlerhood. In addition, these results highlight the importance of task selection when assessing early EF as a predictor of later outcomes, and call for further studies to elucidate the mechanisms through which individual differences in early EF and precursors to EF come about.

## Introduction

Individual differences in executive function (EF) in early childhood have often been shown to be predictive of later academic skills (Blair and Razza, 2007; McClelland et al., 2007; Bull et al., 2008; Clark et al., 2010; Geary et al., 2012). EF refers to a set of cognitive processes needed for goal-directed thought and behavior, and is typically considered to include working memory, inhibition, and shifting (Hughes, 1998; Miyake et al., 2000; Garon et al., 2008). There is now vast evidence that EF predicts mathematics (Bull and Scerif, 2001; St. Clair-Thompson and Gathercole, 2006; Brock et al., 2009; Lee et al., 2012; Van der Ven et al., 2012; Friso-van den Bos et al., 2013), and (early) literacy and reading (Adams and Snowling, 2001; Welsh et al., 2010; Engel de Abreu et al., 2014), both concurrently and over time. Across studies, relationships with EF are generally stronger for mathematics than for literacy and reading (Brock et al., 2009; Willoughby et al., 2012; Fitzpatrick et al., 2014; McClelland et al., 2014; but see Miller et al., 2013).

Most of the earlier work on the predictive value of EF for later academic performance has focused on kindergartners and school-aged children (Blair and Razza, 2007; Mazzocco and Kover, 2007; Bull et al., 2008; Best et al., 2011; Toll et al., 2011; Willoughby et al., 2012). Research on EF in children below age three is relatively scarce. EF typically develops rapidly at this young age (Garon et al., 2013), which might make EF a valuable target for early identification of at-risk children and subsequent interventions. However, the rapid development of EF may imply that EF should not be assessed too early, as the construct might then be unstable.

In the present study, we investigate to what degree individual differences in EF predict later (emergent) academic skills, when EF is assessed at a very young age, that is, in two-year-old children. Recent advances in assessment methods of EF in infants and toddlers enabled us to study EF in such young children, and consequently, begin to explore the predictive value of EF in the first years of life for later (academic) outcomes (Garon et al., 2008, 2013; Mulder et al., 2014; Hendry et al., 2016).

Major advances in assessment methods of EF in very young children have occurred in at least two ways over the past decade. First, an increasing number of EF tests has been designed for children this young (e.g., Hughes and Ensor, 2005; Garon et al., 2008, 2013; Willoughby et al., 2010; Mulder et al., 2014). These tasks are often brief to administer, to make them suitable for infants’ and toddlers whose attention spans are relatively short, and have simple instructions, sometimes accompanied by gestures, to reduce the influence of language skills on task performance. Second, there is increasing awareness amongst researchers that the most reliable measure of EF can be obtained by working with a battery of EF tasks and latent factor modeling, rather than using single task scores (Willoughby et al., 2010; see also Bull and Lee, 2014 for a similar discussion regarding the assessment of EF in older children). Scores on single EF tasks are likely to be strongly confounded with individual differences in motor and language skills, and subject to high measurement error in young children. Such influences are reduced when working with latent factors, particularly if motor and language demands vary between tasks. In support of this, Willoughby et al. (2010) showed that correlations between EF, IQ and ratings of ADHD symptoms were much stronger when working with a latent EF factor compared to working with separate EF task scores in three-year-olds.

### Factor Structure of EF in Early Childhood

Following the seminal work by Miyake et al. (2000), a tripartite distinction in EF is usually made, according to which EF involves three cognitive functions: (i) working memory, or the ability to update information which is stored in memory, (ii) inhibition, or the ability to suppress automatized or predominant responses, and (iii) shifting, or the ability to switch between cognitive sets or tasks. In a recent update of their model, Miyake and Friedman (2012) included a common EF factor, representing shared variance across all EF tasks, and additional specific shifting and working memory factors. In this more recent model, the factor previously labeled inhibition is replaced with the common EF factor.

Studies on the latent factor structure of EF in young children show mixed results, which are likely at least in part due to inter-study variability in the EF measures used across studies (see also Miller et al., 2012). However, a general finding is that EF becomes increasingly differentiated with age. Specifically, in school-aged children, two- or three-factor models of EF, including working memory, inhibition, and/or shifting factors, are often reported (e.g., Huizinga et al., 2006; Lee et al., 2013; Van der Ven et al., 2013). For children below age four, most studies find that different tasks assumed to assess different EF processes typically load onto one single latent EF factor (Wiebe et al., 2008, 2011; Willoughby et al., 2010, but see Hughes, 1998; Miller et al., 2012).

The idea that EF becomes increasingly differentiated with age receives support from studies in which the same EF battery was administered to children of a broad age range. Three such studies have shown that a single latent EF factor fitted the data best up until middle childhood, while multiple latent factors proved a better fit in early adolescence (Shing et al., 2010; Lee et al., 2013; Xu et al., 2013). Thus, notwithstanding mixed findings in earlier work on the factor structure of EF, a relatively robust finding across studies is that EF constitutes one single factor in early childhood, and becomes increasingly differentiated with age.

### EF and (Emergent) Academic Skills in Preschoolers and Kindergartners

A wealth of studies on the relationship between EF and emergent academic skills in preschoolers, kindergartners, and older children has shown that EF significantly relates to both mathematics and literacy skills (e.g., Alexander et al., 1993; Bull and Scerif, 2001; Blair and Razza, 2007; McClelland et al., 2007, 2014; Clark et al., 2010, 2013, 2014; Welsh et al., 2010; Roebers et al., 2012; Shaul and Schwartz, 2014; Bryce et al., 2015). For example, Welsh et al. (2010) investigated whether a composite EF measure at the beginning of preschool (age 4.5 years) predicted growth in literacy and mathematics from beginning to end of preschool in children from low-income families. Indeed, EF significantly predicted growth in both literacy and mathematics over this period, after controlling for individual differences in language ability. Blair and Razza (2007) found that inhibitory control was related to both mathematics and literacy (phonemic awareness and letter knowledge) in kindergarten. Moreover, inhibitory control assessed in preschool predicted mathematics but not literacy in kindergarten, over and above the contribution of inhibitory control in kindergarten. Finally, a meta-analysis by Duncan et al. (2007) highlighted the importance of attention skills in predicting academic achievement even after controlling for children’s prior academic skills, (see Pagani et al., 2010 for similar results). Across studies, the finding that EF predicts academic skills in early childhood appears to be robust.

Two explanations of the associations between EF and academic skills have been proposed (cf. Welsh et al., 2010; Stevens and Bavelier, 2012), which are not necessarily mutually exclusive. First, it has been assumed that EF is directly required for performing academic tasks – that is, there is *task specific* involvement of EF (cf. Blair and Razza, 2007; Bull et al., 2008; Brock et al., 2009). For example, solving mathematical problems likely depends for a substantial part on working memory, in particular, on the retrieval and storage of partial results and processing of information while it is stored (Dehaene, 1997; Cragg and Gilmore, 2014). Hence, children with lower working memory skills may not be able to store and update intermediate results, while working on other parts of a math problem. Similarly, selective attention, an important aspect of EF in early childhood (Garon et al., 2008), has been considered a prerequisite for developing academic skills, as it involves selectively focusing attention on stimuli, such as isolating phonemes from words or focusing on important steps in mathematical problems (for a review, see Stevens and Bavelier, 2012). A second explanation of the relationship between EF and academic skills holds that EF impacts on children’s academic achievement indirectly – that is, *general* involvement of EF is required in (classroom) learning. More specifically, the idea is that well-developed EF skills facilitate behavioral regulation and learning-related behaviors which, in turn, are needed for optimal learning in the classroom. High EF abilities would facilitate children’s ability to pay attention to the teacher’s instruction and could contribute to children’s on-task and goal-directed behavior (Gathercole, 2008; Fitzpatrick and Pagani, 2012), thus allowing them to profit maximally from learning activities (Alexander et al., 1993; Howse et al., 2003; Duncan et al., 2007). In support of this, Nesbitt et al. (2015) found that four-year-olds with higher performance on EF tasks were less frequently disengaged and disruptive, and showed more active participation in the classroom. These behaviors, in turn, were significantly related to children’s emergent academic skills.

A common finding in earlier studies on preschoolers and kindergartners is that EF predicts mathematics more strongly than literacy (e.g., Blair and Razza, 2007; Brock et al., 2009; Willoughby et al., 2012; Fitzpatrick et al., 2014; McClelland et al., 2014, but see Miller et al., 2013). Willoughby et al. (2012), for example, found that a latent EF factor was a strong predictor of a latent academic achievement factor in a large sample of five-year-olds from predominantly low socioeconomic status backgrounds, but significantly more strongly so for mathematics than literacy. Brock et al. (2009) showed that EF predicted mathematics in kindergarten, even after controlling for earlier mathematics scores and general intelligence. In contrast, only earlier reading scores and general intelligence predicted reading scores in kindergarten, and EF did not. Moreover, Fitzpatrick et al. (2014) showed that differences in EF were significantly concurrently related to emergent mathematics and literacy in preschoolers, even after controlling for processing speed and general intelligence. Yet, when controlling for vocabulary, the association with early literacy (i.e., letter-word identification) was no longer significant. McClelland et al. (2014) showed that growth in EF across four measurement waves from prekindergarten to kindergarten predicted growth in mathematics, but not literacy. However, Miller et al. (2013) observed no differential relations between EF and mathematics and literacy in a sample of three- to five-year-olds. In this study, working memory was a unique predictor of mathematics and literacy scores over and above age, inhibition, vocabulary, and social understanding. Thus, with some exceptions, a common finding in earlier early childhood studies is that EF is related to mathematics more strongly than to literacy.

Blair and Razza (2007) proposed that differences in the strength of the relationships between EF and the two academic domains may be due to the differential nature of these domains. In particular, the ability to solve mathematics problems never becomes fully automatized as children grow older, as children need to consider which strategy or rule is most appropriate for each problem, placing relatively strong demands on EF. Solving mathematics problems, or even simple arithmetic tasks, requires one to keep the teachers’ instructions in mind, select a strategy and shift between strategies when necessary, remember the outcome of intermediate computational steps, and ignore distraction (Van der Ven et al., 2012; Bull and Lee, 2014). Just like any learning task, literacy tasks also require one to keep teacher’s instructions in mind and ignore distraction, but these tasks draw less strongly on strategy selection and switching between strategies. Indeed, literacy skills, such as phonemic awareness and letter knowledge, become increasingly automatized, and thus less effortful, as children grow older (cf. Blair and Razza, 2007). At earlier stages, however, EF may be involved in the integration of auditory and visual information and in the automatic retrieval of linguistic information from memory while recognizing sounds and letters (Altemeier et al., 2008). Manipulating speech sounds as in phonemic awareness tasks relies, at least in part, on the ability to selectively attend to speech sounds (cf. Stevens and Bavelier, 2012), and manipulate verbal information while it is stored, such as in sound categorization tasks in which children listen to someone naming three or four pictures (e. g., ball, phone, and bath) and are asked to identify which word does not begin with the same sound as the other two words (Oakhill and Kyle, 2000).

In sum, there is ample evidence that EF is related to academic performance from approximately age three onward. Far less is known about these relations in younger children. To the best of our knowledge, only three studies investigating the predictive effects of EF on later academic performance have included children under age three. In the first study, Fitzpatrick and Pagani (2012) found that working memory performance, averaged across assessments at toddler (29 months) and preschool (41 months) age, predicted number knowledge and receptive vocabulary at age six. The reason for averaging scores across assessments was to reduce the influence of measurement error. In the second study, Merz et al. (2014) found that a broad composite measure of EF in a group of two- to four-year-old children predicted emergent mathematics and literacy a year later, even after controlling for initial performance in these domains. However, the mean assessment age in this study was three years. As such, neither of the studies by Fitzpatrick and Pagani (2012) and Merz et al. (2014) provides insight into the predictive value of EF at toddler age for later academic skills. In a recent study, using data from the same cohort as reported here, we showed that a latent EF factor at two years predicted children’s performance on a latent pre-academic factor one year later (Mulder et al., 2014). This pre-academic factor at three years consisted of early math skills (i.e., a composite score of items assessing number sense, measurement, and geometry, taken from a standardized early math test for toddlers, Op den Kamp and Keuning, 2011) and receptive vocabulary. However, in this study, no distinction was made between emergent mathematics and literacy, and the interval between the two study waves was relatively short. Thus, on the basis of earlier work, it is as yet an open question whether EF in children as young as 2 years of age predicts emergent mathematics and literacy in kindergarten, which, in turn, are predictive of academic performance across elementary school (e.g., Magnuson and Duncan, 2016).

### The Current Study

In the current study, we investigated whether the patterns of relations between EF, literacy and mathematics found in older children, can be found at a younger age than previously investigated. Specifically, our first aim was to investigate whether individual differences in EF in children as young as two years are predictive of emergent mathematics and literacy at age five years. Our second aim was to examine whether EF is a significantly stronger predictor of emergent mathematics than of emergent literacy.

Data from a large longitudinal cohort study were used. In order to reach children from diverse family backgrounds in this study, EF assessments were administered in the field (i.e., in preschool, daycare, or at home) rather than in a lab setting. Given a lack of EF measurement instruments that could be used outside of the lab at the onset of the study, a new battery of EF tasks was developed for field-based administration. This battery has previously been validated for use with two-year-olds (Mulder et al., 2014), and includes a measure of working memory, as well as measures of verbal^1^ and visuospatial short-term memory and selective attention. The latter three are not typically used as indicators of EF in studies of older children, but these are important precursor skills of more complex EF in early childhood (Garon et al., 2008; Hendry et al., 2016).

In order to reduce the influence of measurement error in our assessment of EF, we adopted a latent factor approach (Willoughby et al., 2010, 2012). As our measures assessed precursor skills to more complex EF (i.e., short-term memory and selective attention) as well as a more conventional EF measure (i.e., working memory), we labeled the latent construct ‘early EF,’ for consistency with the early childhood literature (e.g., Hendry et al., 2016) and to differentiate from studies on EF in older children, which typically include only measures of more complex EF’s (i.e., shifting, inhibition, and working memory). Finally, like several other studies on relationships between EF and academic skills (Welsh et al., 2010; Gray et al., 2014), we controlled for children’s receptive vocabulary skills, to rule out that relationships between EF and academic skills found were due to shared variance with vocabulary skills.

## Materials and Methods

### Participants

Data were analyzed from 552 preschool children who were selected from a larger sample participating in the pre-COOL study, a longitudinal study on preschoolers’ cognitive and linguistic development in the Netherlands (see Mulder et al., 2014; Slot et al., 2015, 2017; Verhagen et al., 2017). In pre-COOL, over 3000 children were enrolled. The first and second study wave took place when children were aged two and three years, respectively. These children had been recruited through preschool and daycare centers as well as municipality records (for more details, see Mulder et al., 2014). A sub-group of 751 participants subsequently enrolled in the so-called “core cohort” in kindergarten (study wave three and four, at ages four and five years, respectively).^2^ For the current study, we included children who had enrolled in the pre-COOL study at wave one and had entered the core cohort in kindergarten. Out of all 751 children in the core cohort, 149 had entered the study only at the second wave (age three) due to later enrollment in preschool, and were excluded. A further 50 (8%) children from the remaining 602 children were excluded because they were either older than 36 months (*n* = 4) or younger than 24 months (*n* = 13) at wave one, or because age information was missing (*n* = 33).

The final sample of 552 children included 236 boys [47%, *n* = 44 (8%) gender unknown to the researchers]. Mean age was 29 months at the first study wave (*SD* = 3, range 24–36) and 70 months at the final wave (*SD* = 2, range 64–77). Parents reported on their educational level in questionnaires. If this information was not available, school registry information was used where available. Parental educational level was assessed on a 4-point scale ranging from (1) ‘primary school,’ to (2) ‘lower vocational training,’ (3) ‘secondary school and/or vocational training,’ and (4) ‘higher education (i.e., college or university degree)’, and averaged for both parents. Mean parental educational level was available for *n* = 439 children, with a mean score of 3.10 (*SD* = 0.80, range 1–4, *n* = 35 of 439 (8%) had a mean educational level of 1–1.5; *n* = 67 (15%) had 2–2.5; *n* = 231 (53%) had 3–3.5, *n* = 106 (24%) had 4; *n* = 113 of 552 (21%) missing). Home language was also measured in parent questionnaires. Specifically, parents indicated whether their children were only exposed to Dutch at home, or (also) to another language or multiple other languages. If questionnaire data were missing, research assistants’ reports were used. RA’s were instructed to ask parents and/or teachers at preschool or daycare about the child’s home language background (see also Mulder et al., 2014). The majority of the children (*n* = 363 / 73%, 52 missing) were from monolingual Dutch homes. The remaining children (*n* = 139) were from families in which one or more languages other than Dutch instead of or next to Dutch were spoken.

### Materials

At age two, children were administered a series of tasks assessing EF and precursors to EF (from here on referred to as measures of ‘early EF’ for brevity), and language skills. At age five, they were administered tasks assessing EF and language as well as tasks assessing emergent mathematics and literacy skills. For the current study, data collected with the early EF tasks at age two and the mathematics and literacy tasks at age five were used. In our analyses, receptive vocabulary assessed at age two was used as a control variable. One mathematics task which was administered at the final wave and assessed children’s knowledge of numbers between 1 and 10 was not included in the analysis, because of ceiling performance (see Kolkman et al., 2013 for the same finding with this task in five-year-olds). Regarding early EF, an inhibition task which was included at the first study wave was dropped from the battery after a few 100 children were tested because it turned out to be too difficult (see Mulder et al., 2014), and thus was not included in the current study either. All computerized tasks were programmed in E-prime 2.0 (Schneider et al., 2002).

### Control Measure: Receptive Vocabulary at Two Years

At the first study wave, receptive vocabulary was assessed with a shortened version of the Dutch Peabody Picture Vocabulary Test (PPVT-III-NL, Dunn and Dunn, 2005). In this test, children choose one out of four pictures after an orally presented word. To reduce fatigue, an adapted version was used in which a fixed number of 24 items were presented to all children. Moreover, a laptop was used rather than a test booklet, to facilitate administration and scoring (see Verhagen et al., 2017). Scores were computed as the percentage of correct responses out of the total number of responses for children who responded to at least half of the items of the task (to avoid calculating scores on the basis of few responses). A total of *n* = 527 (95.5%) children obtained a score on the task (*n* = 18 did not do the task at all; *n* = 7 responded to 1–11 items and their data were excluded). The task showed good internal consistency (α = 0.88).

### Early Executive Function Tasks at Two Years

#### Selective Attention

Selective attention was assessed with a visual search task administered on a laptop (Mulder et al., 2014). In this task, children were requested to search for targets (elephants) amongst a display of distractors that were similar in color and size (bears and donkeys). The assessor encouraged the child to search as quickly as possible throughout the task, and provided continuous feedback so that children did not have to remember the rules of the task. That is, if the child pointed to a target, the assessor said: “Well done! Can you find another elephant?”. If the child pointed to one of the distractors, the assessor said: “No, where is an elephant? Try to find the elephants quickly!”. Children were given three practice items, followed by three test items. Each test item consisted of a structured 6 × 8 grid, including eight targets and 40 distractors. Children were allowed to search each display of targets and distractors for 40 s. Item scores were set to missing in cases where the child did not look at the screen at all during these 40 s, according to assessor report (item 1: *n* = 5; item 2: *n* = 4; item 3: *n* = 14). The task score was computed as the average number of identified targets across valid test items for children who responded to at least two items (*n* = 24 children responded to none or only one item and their data were not included). Scores of children who did not find any targets across all test items were set to missing, as we cannot be certain that these children understood the task rules (*n* = 14 children). A total of *n* = 514 (93.1%) children in the current sample obtained a score on the task. The task had good internal consistency (α = 0.86).

#### Visuospatial Working Memory

An adapted version of the Six Boxes task from Diamond et al. (1997) was used to assess visuospatial working memory (see Mulder et al., 2014). In this task, children were shown how six different wooden toys were hidden in six identical white boxes with blue lids. Children were then allowed to search for the toys, by emptying the boxes one at a time. In between search attempts, children were distracted by the assessor for 6 s. As such, after each search attempt, children had to update their memory of which boxes they had already emptied and which boxes still contained a toy, and hold this information in memory over a delay. Following a brief instruction and practice phase, children were allowed six search attempts. Task scores were computed as percentages correct for those children who had searched on all trials.^3^ A total of *n* = 479 (86.8%) children obtained a score on the task (*n* = 30 children did not do the task at all; *n* = 43 searched on 1 to 5 trials and their data were excluded).

#### Visuospatial Short-Term Memory

The visuospatial short-term memory task was based on previous work by Pelphrey et al. (2004) and Vicari et al. (2004) and adapted for the current study (see Mulder et al., 2014). In this task, children were shown how a toy was hidden in one of six identical boxes and asked to search for the toy after a 1-s delay. The task was administered in an adaptive fashion, so that children who passed the one-location item were given a more difficult item in which two toys were hidden in two different locations, etcetera. In the most difficult item, four toys were hidden in four locations. The task score was the highest difficulty level that a child had passed (i.e., number of locations that a child could recall), and could range from zero to four. A total of *n* = 457 (82.8%) children obtained a score on the task.^4^

#### Verbal Short-Term Memory

Verbal short-term memory was assessed with a non-word repetition task (Verhagen et al., 2017). This task contained 2 practice items and 12 test items, half of which were monosyllabic and the other half bisyllabic. The items had been prerecorded in a high-pitch child-friendly voice from a Dutch native speaker and they were presented to the children over headphones. For each test item, children were presented with a short video clip showing a picture of a novel object that appeared out of a drawing of a box. At the same time, they heard a prerecorded sentence that encouraged them to repeat the non-word: “Look, a [keupun]! Say [keupun]!” Children’s repetition attempts were scored online by the assessors as correct, incorrect, or ‘unknown’ (<2% of all responses). Task scores were computed as the percentage of correct responses out of all responses for children who responded to at least half of the items of the task. A total of *n* = 414 (75.0%) children obtained a score on the task (*n* = 83 did not do the task at all; *n* = 55 responded to 1–5 items and their data were excluded). The task showed good internal consistency (α = 0.86).

### Emergent Mathematics at Five Years

#### Number Knowledge (1–100)

A number naming task was used to assess number knowledge (Kolkman et al., 2013). In this task, children were presented with written numerals on a laptop screen and asked to name each numeral. The numerals presented were in the 1–100 range. The task contained five test items (i.e., 12, 30, 54, 70, and 97). Scores were computed as the percentage of correct responses for each child. A total of *n* = 514 children (93.1%) children obtained a score on the task, and all children had responded to all items. The test had good internal consistency (α = 0.81).

#### Number Estimation (1–10)

To assess children’s number estimation skills, a number line task was presented in which children were asked to estimate the position of a given number (in the range from 1 to 10) on a horizontal line (Siegler and Opfer, 2003, current task adapted from Kolkman, 2013, Unpublished). This line was presented on a laptop screen, with ‘1’ and ‘10’ on either side. Prior to the task, children were shown the positions of both extremes, as indicated by ‘1’ and ‘10.’ They were then asked to locate the position of a given number on the line. The task contained six test items. Linear fit scores were obtained by calculating the squared correlation between children’s responses and the values corresponding to the location of the numbers on the number line (Geary et al., 2008). Linear fit scores have been shown to be a valid measure of number mapping in young children (Friso-van den Bos et al., 2014). A total of *n* = 515 children (93.3%) children obtained a score on the task, all of whom had responded to at least five items.

#### Number Estimation (1–100)

To investigate children’s number estimation of higher numbers, a number line task was presented which was the same as the previous one, except that numbers between ‘1’ and ‘100’ were presented (adapted from Kolkman, 2013, Unpublished). This task contained six test items. As in the number line 1–10 task, linear fit scores were computed. A total of *n* = 513 children (92.9%) children obtained a score on the task, and all had responded to at least five items.

#### Cito Mathematics

Mathematical abilities were measured with the criterion-based Cito Mathematics Test for Kindergarteners (Janssen et al., 2005, 2010). Cito Mathematics tests are part of the student achievement monitoring system used in most Dutch primary schools. The earliest Cito assessments take place in the kindergarten departments of primary school, at ages four and five. The version used in this paper was administered mid-year 2 of kindergarten and contained 54 items that were administered on two separate days. Three main domains were covered by the test: (a) number knowledge (e.g., recognizing numbers), (b) measurement (e.g., weight and length), and (c) geometry (e.g., shapes and figures). Raw scores were converted into Rasch-based ability scores (Janssen et al., 2005) that can be directly compared across kindergarten and the primary school period. Scores were available for *n* = 419 children (75.9%). The reliability coefficient of the version used mid-year 2 is 0.87 (Koerhuis and Keuning, 2011).

### Emergent Literacy at Five Years

#### Letter Knowledge

Letter knowledge was assessed with a shortened version of the letter recognition task used in De Jong (2007). In this task, children were presented with a laminated sheet of paper on which eight letters in lowercase were presented. The assessor then provided children with a certain letter (pronounced phonetically) and asked children to point to the correct letter on the sheet. Scores were computed as the percentage of letters identified correctly out of all responses. A total of *n* = 499 (90.4%) children obtained a score on the task (*n* = 35 did not do the task at all; *n* = 18 responded to 1–4 items and their data were excluded). Internal consistency of the task was sufficient (α = 0.79).

#### Phonemic Awareness

A first sound awareness task was used to assess phonemic awareness, which was taken from De Jong (2007). In this task, children were presented with an array of four pictures presented on a laptop screen. One of these pictures was marked as the target picture. The child’s task was to identify the first sound of the word describing the target picture and determine which of the three other pictures displayed a word starting with the same first sound. Children were presented with a relatively long instruction that became shorter after the first four test items. Specifically, for each of the first four items of the task, the assessor named the target picture (e.g., ball) and told children the first sound of this word (/b/). The assessor then also named the three other pictures (e.g., bear, doll, and phone) and asked the child to indicate which picture was labeled with a word starting with the same first sound as the label of the target picture (in this case: bear). From the fifth item onward, the assessor no longer named the first sound of the word describing the target picture, but asked directly which of the three other pictures represented a word starting with the same sound. The task contained two practice items and 12 test items. Scores were calculated as the percentage of correct responses out of all responses for children who responded to at least half of the task. A total of *n* = 497 (90.0%) children obtained a score on the task (*n* = 38 children did not do the task at all; *n* = 17 children responded to 1–5 items and their data were excluded). Internal consistency of the task was good (α = 0.84).

#### Cito Language and Literacy

General language and literacy skills were measured by the Cito Language Test for Kindergartners (Lansink, 2009). This is a standardized national test that, like the Cito Mathematics test described above, is part of the student achievement monitoring system commonly used in primary schools in the Netherlands. The test administered at mid-year 2 in kindergarten contained 60 items, which were administered on two separate days. The items covered two broad domains: (a) conceptual awareness and (b) language awareness. The conceptual domain was assessed with items testing children’s receptive vocabulary and listening skills. The emergent literacy domain was assessed with items testing children’s sound and rhyme awareness, hearing first and last words in sentences, auditory synthesis, and letter recognition. Scores were available for *n* = 414 children (75.0%). Raw scores were converted into Rasch-based ability scores. Previous studies show good internal consistency of the test (α = 0.89, Lansink and Hemker, 2012).

### Procedure

Tasks were administered by trained research assistants in a quiet room at children’s homes or preschools/daycare (age two years) or at their schools (age five years). At age two, the tasks were intermixed with four other tasks not reported in the current paper^5^, and presented in the following fixed order: receptive vocabulary, selective attention, verbal short-term memory, visuospatial short-term memory, visuospatial working memory. At age five, tasks were intermixed with nine other tasks^6^, and presented in the following order: letter knowledge, number naming and number line tasks, and phonemic awareness. Both sessions lasted about 45 min. To make sure that assistants adhered to the standardized procedures of each task, they had undergone an intensive training prior to data collection, which involved a full-day training, administration of a video recording of a session with a child of the relevant age, and elaborate feedback reports and discussion (for further details, see Mulder et al., 2014). The Cito tests were administered by children’s teachers, following a standardized protocol.

### Analyses

Since children differed substantially in age at the time of data collection, and development is rapid at this age, all early EF, literacy and mathematics measures were corrected for age. However, especially at age two years, children from higher SES backgrounds were tested at a younger age than children with lower SES backgrounds, due to the sampling procedures used in pre-COOL. Since SES was also positively related to most cognitive measures (early EF, mathematics, and literacy), this confound entailed that merely taking age-residualized scores would give an underestimate of the true effect of age (a suppressor effect). In order to counter this suppressor effect, regression analyses were run for each variable with both age at the time of that particular measure and parental education as predictors, and residual scores were determined based on only the age coefficient in this analysis (as correcting for SES would yield the undesired effect of correcting for a source of genuine variation in early EF). The correlations between the original variables and the corrected variables ranged from *r* = 0.895 to *r* = 0.994 (mean *r* = 0.980).

The analyses were run using the Lavaan package of the statistical software R (Rosseel, 2012). In all analyses, full information maximum likelihood estimation with robust (Huber-White) standard errors was used to handle missing data. Model fit was evaluated on the basis of the following commonly used cut-off criteria: RMSEA < 0.05, CFI > 0.90, and SRMR < 0.08 (Hu and Bentler, 1999). The chi-square index was not used, since it is very sensitive to sample size and typically significant in large samples (Little, 2013; Brown, 2015).

The analyses were performed in three steps. First, a Confirmatory Factor Analysis (CFA) was performed to see whether the tasks assessing emergent mathematics and literacy indeed represented two separate latent factors. To this aim, a two-factor model was fitted in which the mathematics tests loaded on one factor and the literacy tests on another factor. In this model, the Cito mathematics and Cito literacy tests were correlated, to deal with shared method-bound variance (Brown, 2015).

Second, for our main analysis concerning whether early EF at age two predicted emergent mathematics and literacy at age five, a Structural Equation Model (SEM) was fitted in which early EF, as a latent factor and based on all four tasks, predicted the two latent factors mathematics and literacy. In this model, the paths from early EF to mathematics and from early EF to literacy were freely estimated. Receptive vocabulary, home language and parental education were included as control variables.

Finally, to test our prediction that the effect of early EF on mathematics would be stronger than the effect on literacy, we fitted a second model in which the paths from early EF to mathematics and from early EF to literacy were constrained to be equal, rather than freely estimated. Fit of the model in which the paths were freely estimated and the model in which these paths were constrained was then compared through a chi-square difference test. If the outcome of this test was significant, the less constrained model was the preferred model; if non-significant, the more constrained (more parsimonious model) was the preferred model.

## Results

### Descriptives and Correlations

Descriptive statistics and correlations for all tasks are provided in **Tables 1, 2**, respectively.

**Table 1:** Descriptive statistics for all tasks.

	*M*	*SD*	Range	Skew (SE)	Kurtosis (SE)	*N* (% completed)
**Age 2**					
*Early EF*						
Selective attention	3.66	1.67	0.33–7.67	–0.15 (0.11)	–0.69 (0.22)	514 (93.12%)
Visuospatial working memory	65.76	19.24	0–100	–0.31 (0.11)	–0.08 (0.22)	479 (86.78%)
Visuospatial short-term memory	2.01	0.91	0–4	0.37 (0.11)	–0.37 (0.23)	457 (82.79%)
Verbal short-term memory	41.44	27.84	0–100	0.29 (0.12)	–0.86 (0.24)	414 (75.00%)
*Control variable*						
Receptive vocabulary	63.42	19.71	8.33–100	–0.21 (0.11)	–0.73 (0.21)	527 (95.47%)
**Age 5**					
*Emergent mathematics*						
Number line 1–10	0.85	0.21	0–1	–2.58 (0.11)	6.44 (0.22)	515 (93.30%)
Number line 1–100	0.51	0.33	0–0.99	–0.18 (0.11)	–1.41 (0.22)	513 (92.93%)
Number knowledge 1–100	76.33	18.38	11.11–100	–0.52 (0.11)	0.01 (0.22)	514 (93.12%)
Cito mathematics	84.46	12.05	49–135	0.56 (0.12)	1.43 (0.24)	419 (75.91%)
*Emergent literacy*						
Phonemic awareness	81.02	22.53	8.33–100	–1.29 (0.11)	0.92 (0.22)	497 (90.04%)
Letter knowledge	81.96	24.13	0–100	–1.32 (0.11)	0.94 (0.22)	499 (90.40%)
Cito language and literacy	66.11	10.02	41–108	0.43 (0.12)	0.66 (0.24)	414 (75.0%)

**Table 2:** Zero-order correlations for all tasks.

	1	2	3	4	5	6	7	8	9	10	11
**Early EF at age 2**
(1) Selective attention	–	0.22^∗∗∗^	0.24^∗∗∗^	0.18^∗∗∗^	0.13^∗∗^	0.13^∗∗^	0.20^∗∗∗^	0.28^∗∗∗^	0.07	0.14^∗∗^	0.21^∗∗∗^
(2) Visuospatial WM	0.17^∗∗∗^	–	0.17^∗∗∗^	0.18^∗∗∗^	0.11^∗^	0.13^∗∗^	0.12^∗^	0.16^∗∗^	0.10^∗^	0.05	0.15^∗∗^
(3) Visuospatial STM	0.19^∗∗∗^	0.13^∗∗^	–	0.16^∗∗^	0.12^∗^	0.05	0.12^∗^	0.14^∗^	0.13^∗∗^	0.15^∗∗^	0.10
(4) Verbal STM	0.16^∗∗^	0.16^∗∗^	0.15^∗∗^	–	0.17^∗∗^	0.03	0.13^∗^	0.18^∗∗^	0.22^∗∗∗^	0.14^∗∗^	0.20^∗∗∗^
**Emergent mathematics at age 5**	
(5) Number line 1–10	0.16^∗∗∗^	0.13^∗∗^	0.13^∗∗^	0.18^∗∗∗^	–	0.22^∗∗∗^	0.35^∗∗∗^	0.21^∗∗∗^	0.26^∗∗∗^	0.29^∗∗∗^	0.20^∗∗∗^
(6) Number line 1–100	0.18^∗∗∗^	0.14^∗∗^	0.06	0.04	0.21^∗∗∗^	–	0.36^∗∗∗^	0.30^∗∗∗^	0.24^∗∗∗^	0.26^∗∗∗^	0.26^∗∗∗^
(7) Number knowledge 1–100	0.26^∗∗∗^	0.13^∗∗^	0.12^∗^	0.13^∗∗^	0.34^∗∗∗^	0.35^∗∗∗^	–	0.42^∗∗∗^	0.39^∗∗∗^	0.50^∗∗∗^	0.33^∗∗∗^
(8) Cito mathematics	0.34^∗∗∗^	0.16^∗∗^	0.14^∗∗^	0.19^∗∗∗^	0.22^∗∗∗^	0.29^∗∗∗^	0.41^∗∗∗^	–	0.33^∗∗∗^	0.33^∗∗∗^	0.66^∗∗∗^
**Emergent literacy at age 5**
(9) Phonemic awareness	0.15^∗∗^	0.13^∗∗^	0.15^∗∗^	0.23^∗∗∗^	0.25^∗∗∗^	0.24^∗∗∗^	0.38^∗∗∗^	0.33^∗∗∗^	–	0.53^∗∗∗^	0.42^∗∗∗^
(10) Letter knowledge	0.17^∗∗∗^	0.05	0.14^∗∗^	0.14^∗∗^	0.28^∗∗∗^	0.25^∗∗∗^	0.49^∗∗∗^	0.33^∗∗∗^	0.52^∗∗∗^	–	0.31^∗∗∗^
(11) Cito language	0.29^∗∗∗^	0.17^∗∗^	0.12^∗^	0.22^∗∗∗^	0.22^∗∗∗^	0.26^∗∗∗^	0.33^∗∗∗^	0.65^∗∗∗^	0.42^∗∗∗^	0.31^∗∗∗^	–

*^∗∗∗^*p* < 0.001, ^∗∗^*p* < 0.01, ^∗^*p* < 0.05. Correlations above the diagonal are based on true task scores, correlations below the diagonal are based on age-residualized scores.*

### Confirmatory Factor Analyses

The outcomes of the CFA in which a two-factor model was estimated, containing an emergent mathematics and an emergent literacy factor, showed good data fit, RMSEA = 0.04, CFI = 0.99, SRMR = 0.03 (*n* = 530). The correlation between the latent factors ‘mathematics’ and ‘literacy’ in this model was 0.58 (*p* < 0.001), and the correlation between the error terms of the two Cito tests was 0.83. The model fitted significantly better than a model in which all tasks loaded on a single latent academic factor, Δχ^2^(1) = 29.14, *p* < 0.001 (model fit of the one-factor model: RMSEA = 0.07, CFI = 0.96, SRMR = 0.03).

### Relationships between Early EF at Two and Emergent Mathematics and Literacy at Five

**FIGURE 1:**
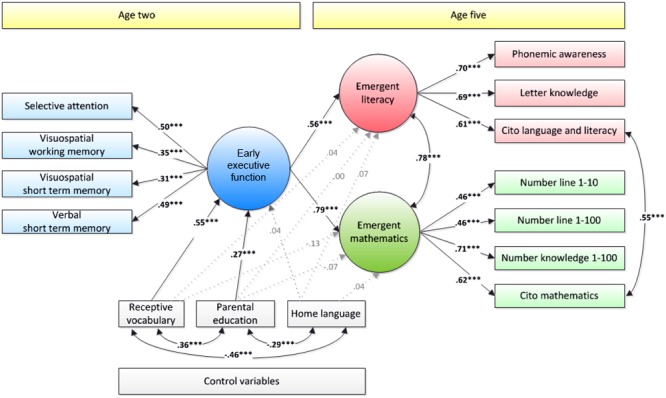
Structural Equation Model with early executive function (EF) at age two as a predictor of emergent mathematics and literacy at age five. Standardized estimates are presented. Dotted, gray lines represent non-significant coefficients. ^∗^*p* < 0.05, ^∗∗^ *p* < 0.01, ^∗∗∗^*p* < 0.001.

The SEM model in which the latent factor early EF was modeled as a predictor of the latent factors emergent mathematics and literacy, with parental education, home language, and receptive vocabulary at age 2 controlled, fitted the data well, RMSEA = 0.05, CFI = 0.93, SRMR = 0.05 (*n* = 552). This model is depicted in **Figure 1**.

As can be seen in this figure, the model showed positive and significant relationships from early EF to emergent literacy and from early EF to emergent mathematics, after controlling for receptive vocabulary, parental education, and home language. These associations were positive and strong for both factors: β = 0.56, *p* < 0.001 for emergent literacy; β = 0.79, *p* < 0.001 for emergent mathematics.

To test whether the association between early EF and mathematics was stronger than the association between early EF and literacy, an alternative model was fitted in which the paths from early EF to literacy and from early EF to mathematics were constrained to be equal instead of freely estimated. This alternative model fitted the data slightly less well, as indicated by the absolute fit indices of this model, RMSEA = 0.05, CFI = 0.92, SRMR = 0.06, than the previous, less constrained model. However, the result of a chi-square difference test showed that the difference in fit of the two models approached significance, but did not surpass the 0.05 alpha level, Δχ^2^(1) = 3.14, *p* = 0.076. Thus, the more parsimonious model in which both paths were constrained to be equal was the preferred model. This indicates that there was no significant difference in the strength of the relationship with early EF between both emergent academic skills. The size of the two constrained relationships was estimated at *B* = 0.63 (β = 0.68 for mathematics and β = 0.67 for literacy, *p* < 0.001).

A possible reason for why no clear difference in the strength of the associations was found, was that unlike in some of the earlier studies described above (Brock et al., 2009; Willoughby et al., 2012; Fitzpatrick et al., 2014), in our study, a verbal memory indicator of early EF was included (i.e., non-word repetition). Non-word repetition is known to be a strong predictor of children’s later language and literacy skills, in particular, letter knowledge (De Jong and Olson, 2004) and reading (Gathercole et al., 1991; Kibby et al., 2014). This might have strengthened the relationship between early EF and literacy.

**FIGURE 2:**
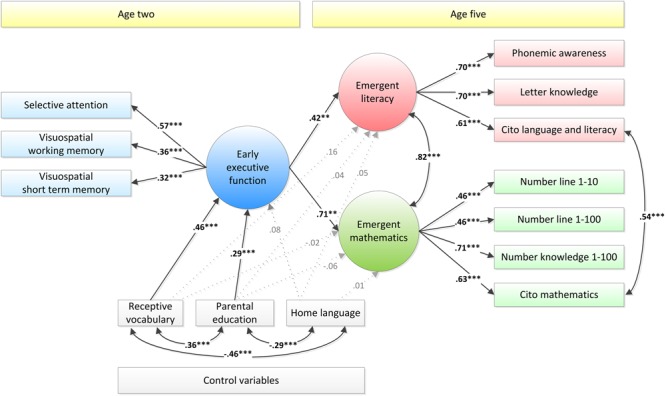
Structural Equation Model with early EF at age two as a predictor of emergent mathematics and literacy at age five, with only non-verbal tasks as indicators of early EF. Standardized estimates are presented. Dotted, gray lines represent non-significant coefficients. ^∗^*p* < 0.05, ^∗∗^*p* < 0.01, ^∗∗∗^*p* < 0.001.

To examine this possibility, we ran an additional analysis in which we fitted a model that was the same as the model depicted in **Figure 1**, except that non-word repetition was removed as an indicator of early EF, such that only non-verbal measures of early EF remained. The resulting model, which is presented in **Figure 2**, showed good data fit, RMSEA = 0.05, CFI = 0.93, SRMR = 0.05.

As shown in **Figure 2**, the association between early EF and emergent literacy in this adapted model was weaker than in the previous model, albeit still significant: β = 0.42, *p* = 0.010 rather than β = 0.56, *p* < 0.001. The association between early EF and emergent mathematics was also weaker than in the previous model, but did not decrease as strongly as the association between early EF and emergent literacy: β = 0.71, *p* = 0.005 rather than β = 0.79, *p* < 0.001. All other coefficients in the model were comparable in size to those in the previous model. A comparison with a model in which the paths from EF to mathematics and literacy were constrained showed that the unconstrained model fitted the data significantly better than the constrained model, Δχ^2^(1) = 10.13, *p* = 0.001. This indicates that the relationship between early EF and mathematics was significantly stronger than the relationship between early EF and literacy, at least when only non-verbal indicators of the latent early EF construct were included.

## Discussion

In this study, we investigated whether early EF in two-year-olds predicted emergent literacy and mathematics three years later. The results showed significant associations between early EF, treated as a latent factor, and emergent academic performance, in line with earlier research findings (Espy et al., 2004; Blair and Razza, 2007; McClelland et al., 2007; Bull et al., 2008; Clark et al., 2010). The current findings extend previous research in two ways. First, they show that early EF, assessed in children as young as two years, is predictive of emerging academic skills at the end of kindergarten. Second, they indicate that differences in early EF are particularly predictive of emergent mathematics, but also play a role in the development of early literacy skills.

The finding that early EF was a stronger predictor of mathematics than literacy was influenced by the specific tasks used as indicators to our latent early EF construct. Specifically, when we included a non-word repetition task, which required children to process, store, and reproduce non-words, the strength of the association between early EF and mathematics was not significantly different from the strength of the association between early EF and literacy, although a trend toward significance was observed. When this indicator was dropped, and only visuospatial and non-verbal early EF tasks were included, early EF related significantly more strongly to mathematics than literacy. This finding suggests that the specific tasks used to assess EF may explain at least in part some of the mixed results in earlier studies regarding the strength of the associations between EF and mathematics and literacy. More precisely, previous studies reporting differential associations between EF and these two types of academic skills in preschoolers and kindergartners often included EF measures that were either largely non-verbal (Brock et al., 2009) or measures that required children to produce verbal responses (i.e., Willoughby et al., 2012; Fitzpatrick et al., 2014) rather than measures assessing verbal EF skills such as our non-word repetition task (but see McClelland et al., 2014). In two of the studies that did not find stronger relations between EF and emergent mathematics than between EF and literacy in early childhood, verbal working memory tasks, similar to the current study, were included (Welsh et al., 2010; Miller et al., 2013). A wealth of cross-sectional studies has shown that verbal memory is an important predictor of later literacy and reading (for a review, see Swanson et al., 2009). Hence, it is not surprising that, in our study, adding this measure resulted in a stronger relationship between the latent early EF factor and literacy.

A further possible reason why we found stronger relationships between early EF and mathematics than between early EF and literacy is that two out of our early EF tasks assessed spatial skills, that is, visuospatial short-term memory and visuospatial working memory. Earlier research has found clear connections between spatial skills and math abilities (Casey et al., 1995; Gunderson et al., 2012; Mix and Cheng, 2012; Verdine et al., 2014). Mix and Cheng (2012) found, for instance, that training on a mental rotation task enhanced six- to eight-year-olds’ performance on calculation problems.

Thus, our data suggest that the specific tasks used to assess EF may have implications for the predictive associations found between EF and different types of academic skills, with the distinction between verbal and non-verbal EF tasks perhaps playing an important role. Note that, given that the model with the non-word repetition task as indicator to the latent early EF factor showed a non-significant trend effect for differential relations between early EF and literacy as opposed to mathematics, these results need to be interpreted with caution. Further research, in which the indicators to a latent (early) EF construct are varied more systematically, is needed to investigate in more detail how the choice of tasks influences the strength of associations with different academic skills in young children.

A consistent finding in our study – regardless of whether the verbal memory task was included – was that individual differences in early EF at age two significantly predicted children’s emergent mathematics and literacy skills three years later. Theoretically, these associations could either be direct, through children’s reliance on executive processes when performing academic tasks, as argued above, or indirect, through other mediating factors, in particular, children’s ability to regulate their behavior or other learning-related skills needed in order to benefit from instructions in the classroom. Supporting this latter notion, we found earlier that early EF at age two positively predicts self-regulation behavior in the classroom at age three in a subsample of the children investigated in the current study (Slot et al., 2017). Likewise, Nesbitt et al. (2015) have shown that the association between EF and emergent academic skills from the beginning (age four years) to end of pre-kindergarten was mediated by learning-related behaviors, although direct effects between EF and academic skills remained significant when learning-related behaviors were taken into account. Not all studies support the idea that learning-related skills mediate the relationship between EF and academic performance, however. In a study on kindergartners, Brock et al. (2009), for example, did not find that learning-related behaviors were a significant mediator between EF and emergent mathematics. Therefore, these authors concluded that EF was directly involved in academic task performance. Summarizing, although there is some evidence that behavioral regulation and learning-related skills mediate the relationship between EF and academic skills in young children, at least part of the association between EF and academics appears to be direct.

A number of issues need to be taken into consideration when interpreting our findings, in particular regarding the assessment of toddler EF. In our study, we modeled early EF as a single latent factor. The advantage of this approach is that the impact of measurement error is reduced, and the predictive value of latent EF constructs is typically stronger than when single task scores are used in the analysis (cf. Willoughby et al., 2012). Indeed, in our study, associations between the latent early EF factor and the two emergent academics factors in our SEM model were much stronger than the correlations between true task scores. Also, factor loadings to the latent early EF factor were all satisfactory to good, while correlations between the task scores of the early EF tasks were pretty low. This underscores the importance of modeling EF as a latent factor, especially at this young age. However, modeling EF as a latent factor leaves unanswered the question as to which specific EF skills are predictive of later academic skills. Moreover, with respect to the early EF assessment, we included both conventional EF measures, such as working memory, and measures of skills that are seen as important precursor skills to EF in early childhood, that is, selective attention and short-term memory (Garon et al., 2008; Hendry et al., 2016). Ideally, a battery of more complex EF tasks would have been used, involving also inhibitory control and shifting. At the outset of the current study, however, such measures were not available for large scale field-based assessments. More recently, EF batteries for toddlers have been developed that include inhibition and shifting measures (Garon et al., 2013), enabling a more comprehensive assessment of EF in very young children.

Another limitation of the current study is the lack of statistical control for early emerging mathematics and literacy at two years. Some studies have worked with such rigorous controls in slightly older children (e.g., Brock et al., 2009). Although toddlers have been shown to have some basic understanding of numerical transformations (Sophian and Adams, 1987), to the best of our knowledge, no suitable measures of precursors to mathematical abilities were available for field-use in our two-year assessment. In fact, we piloted with a magnitude comparison task for toddlers, but were concerned about its validity for this young age group. Instead, we controlled for vocabulary as a key cognitive factor in toddlerhood. The specific set of statistical controls used when testing associations between EF and academics varies widely between studies, and choices regarding these controls may clearly impact on whether or not differential relations between EF and mathematics vs. EF and literacy are found (see Fitzpatrick et al., 2014). In the current study, for example, including a vocabulary measure, but not a measure of precursors to mathematics skills may have affected our finding that early EF at two years was more strongly predictive of mathematics than literacy at five. Thus, future studies investigating toddler EF as a predictor of achievement should ideally consider including basic tests of literacy and numeracy at this young age already, to provide a stronger test of the independent contribution of EF to future academic attainment.

In addition to including early mathematics and literacy measures as statistical controls in the study of EF as predictor of academic achievement, inclusion of such measures as well as later EF would allow to study the reverse relationship as well: do early literacy and mathematics predict later EF? Clearly, the current study findings do not allow us to draw conclusions regarding the direction of effects between early EF and emergent academics. Recent work shows that associations between EF and mathematics may be bidirectional (for a review, see Clements et al., 2016). For example, Welsh et al. (2010) found that emergent numeracy skills at the beginning of pre-kindergarten predicted EF at the end of pre-kindergarten, while the opposite was also true. In this study, emergent literacy did not predict EF over time. Clements et al. (2016) speculate that high- quality mathematics curricula in particular, may provide optimal situations for scaffolding learning of *both* mathematics and EF in young children.

## Conclusion

The current longitudinal study is the first to investigate the predictive value of early EF in two-year-olds for emerging academic skills over a three-year time interval. The results showed that early EF at two years predicts emergent literacy and mathematics just prior to school entry, after controlling for receptive vocabulary, parental education, and home language. This suggests that early EF can be reliably assessed at this young age, despite the rapid dynamic nature of development during this phase, and has important predictive value for academic achievement three years later. Further work could investigate whether EF measures in toddlerhood can accurately identify children at risk for significant learning impairment in school, and could be implemented as effective screening tools to help identify which children should be referred for intervention. Moreover, findings call for future studies to unravel which genetic and environmental factors impact on early individual differences in EF in the first years of life. Finally, future studies could assess whether individual differences in EF at a very young age affect not only the level but also the growth of children’s academic skills over kindergarten or even elementary school.

## Ethics Statement

Approval for the study was obtained from both Ethical Advisory Committees of the Faculty of Social and Behavioral Sciences of Utrecht University and the Department of Education of the University of Amsterdam. Children were recruited in two different ways, as reported in more detail in Mulder et al. (2014). In short, part of the sample was recruited via directly approaching children’s parents (home-based sample), while another part was recruited through children’s daycare centers and preschools (center-based sample). In the center-based sample, parents were given an information letter in which they were given the opportunity to withdraw their child from participation. In addition, children’s teachers were requested to inform parents about the study and assessments. Passive parental consent was allowed by the Ethical Advisory Committee of the Department of Education of the University of Amsterdam (the institution responsible for data collection), because of the major challenges involved with obtaining permission from all parents, and because all the assessments were child-friendly and non-invasive.

## Author Contributions

HM and JV: task design or adaptation (EF at two years, academic tasks at five years, with the exception of the CITO measures), wrote the manuscript, interpreted the results. SV: analyzed the data, interpreted the results, and revised the manuscript. PS: interpreted the results and revised the manuscript. PL: principal investigator, design of the study, and revised the manuscript.

## Conflict of Interest Statement

The authors declare that the research was conducted in the absence of any commercial or financial relationships that could be construed as a potential conflict of interest.

## Footnotes

1 The verbal short-term memory task was not included in our original psychometric investigation (Mulder et al., 2014). We included this task in the current study to obtain a more balanced mix of early EF tasks, with both verbal and non-verbal measures. To investigate the effect of the inclusion of this task, we conducted our analyses with and without this indicator of the early EF construct.

2 The main criteria for inclusion in the core cohort were the following: (i) children had obtained a test score on the vocabulary and attention tasks, as well as on at least two other tasks of the language and executive function test battery at ages two and/or three years, and (ii) contact information about children’s schools was available. The rationale behind these criteria was to obtain a dataset for the children in the core cohort that was as complete as possible in order to be able to address the main question guiding the pre-COOL study.

3 This relatively stringent criterion was chosen because search attempts were interdependent: that is, each next attempt was more difficult than the previous (successful) attempt, because children had to keep an increasing number of empty boxes in mind as they progressed through the task.

4 The relatively high number of missing on this task was due to the fact that we had to shorten the test after the first few months of data collection, to reduce overall testing time, and data of the children tested on the initial version of the task could not be included (see also Mulder et al., 2014).

5 These four additional tasks assessed children’s phonological abilities, sentence comprehension, and delay of gratification. Task order of all tasks as follows: phonological processing, receptive vocabulary, selective attention, verbal short-term memory, sentence comprehension, delay of gratification (snack delay), visuospatial short-term memory, visuospatial working memory, and delay of gratification (gift delay).

6 These additional tasks measured rapid automatized naming, receptive vocabulary, verbal short-term memory, visuospatial short-term memory, visuospatial working memory, sentence comprehension, inhibition, verbal working memory, and delay of gratification. Task order of all tasks as follows: rapid automatized naming, receptive vocabulary, selective attention, verbal short-term memory, visuospatial short-term memory, visuospatial working memory, sentence comprehension, letter knowledge, inhibition, number naming 1–10, number line 1–10, number naming 1–100, number line 1–100, verbal working memory, phonemic awareness, delay of gratification.
